# Whole Genome Sequencing to Investigate the Emergence of Clonal Complex 23 *Neisseria meningitidis* Serogroup Y Disease in the United States

**DOI:** 10.1371/journal.pone.0035699

**Published:** 2012-04-27

**Authors:** Mary G. Krauland, Julie C. Dunning Hotopp, David R. Riley, Sean C. Daugherty, Jane W. Marsh, Nancy E. Messonnier, Leonard W. Mayer, Hervé Tettelin, Lee H. Harrison

**Affiliations:** 1 Infectious Diseases Epidemiology Research Unit, School of Medicine and Graduate School of Public Health, University of Pittsburgh, Pittsburgh, Pennsylvanian, United States of America; 2 The Institute for Genome Sciences, University of Maryland School of Medicine, University of Maryland, Baltimore, Maryland, United States of America; 3 Meningitis and Vaccine Preventable Diseases Branch, Centers for Disease Control and Prevention, Atlanta, Georgia, United States of America; Health Protection Agency, United Kingdom

## Abstract

In the United States, serogroup Y, ST-23 clonal complex *Neisseria meningitidis* was responsible for an increase in meningococcal disease incidence during the 1990s. This increase was accompanied by antigenic shift of three outer membrane proteins, with a decrease in the population that predominated in the early 1990s as a different population emerged later in that decade. To understand factors that may have been responsible for the emergence of serogroup Y disease, we used whole genome pyrosequencing to investigate genetic differences between isolates from early and late *N. meningitidis* populations, obtained from meningococcal disease cases in Maryland in the 1990s. The genomes of isolates from the early and late populations were highly similar, with 1231 of 1776 shared genes exhibiting 100% amino acid identity and an average π_N_  =  0.0033 and average π_S_  =  0.0216. However, differences were found in predicted proteins that affect pilin structure and antigen profile and in predicted proteins involved in iron acquisition and uptake. The observed changes are consistent with acquisition of new alleles through horizontal gene transfer. Changes in antigen profile due to the genetic differences found in this study likely allowed the late population to emerge due to escape from population immunity. These findings may predict which antigenic factors are important in the cyclic epidemiology of meningococcal disease.

## Introduction

*Neisseria meningitidis* is a leading cause of bacterial meningitis world-wide [1]. The most common disease-causing serogroups are A, B, C, X, W-135, and Y. In the United States, serogroup Y *N. meningitidis* was responsible for an increasing proportion of all meningococcal disease during the 1990s and also for an increased incidence of disease. For example, during 1989–1991, ∼ 2% of invasive meningococcal strains in the U.S. were serogroup Y, whereas by the mid 1990s, over a third of cases were caused by this serogroup. The predominant genetic lineage of serogroup Y isolates from this time period was the ST-23 clonal complex [2], [3], [4].

In a previous study, we demonstrated that the emergence and maintenance of ST-23 complex serogroup Y meningococcal disease in Maryland was associated with antigenic shift in three key meningococcal outer membrane proteins (OMPs), PorA, FetA, and PorB. A change in the pulsed field gel electrophoresis (PFGE) profile was also described, indicating that the strain that predominated in the early 1990s (early strain type) was replaced by another serogroup Y ST-23 complex strain that emerged later in the same decade (late strain type) through clonal replacement [2], [5].

In clonal replacement, one circulating strain is supplanted by another. This phenomenon has been observed in disease-causing strains of *N. meningitidis* [6], in *N. meningitidis* carriage isolates [7], [8], and other bacteria [9]. Clonal replacement may be the result of introduction of new genetic lineages that are more fit, more virulent, more transmissible or allow escape from population immunity due to differences in antigenic proteins. Alternatively, mutation or recombination within a population may result in the development of a sub-population that has distinct genetic features with enhanced fitness or with antigens that have changed enough to allow escape from population immunity. A modeling approach has demonstrated that infectious pathogens may develop stable co-existing populations which have non-overlapping repertoires of dominant antigens even when recombination is a common feature, as a result of selection due to immunity in the host population [10]. This concept would explain the existence of two populations of antigenically different but otherwise genetically similar strains causing disease in the same host population at the same time.

We used whole genome sequencing to more extensively investigate the genetic differences in early and late strain type serogroup Y ST-23 complex *N. meningitidis* isolates obtained from meningococcal disease cases in Maryland during the 1990s. We hypothesized that an accumulation of antigenic differences within an otherwise stable genome was responsible for the emergence of the late ST-23 complex strain type. The increase in serogroup Y meningococcal disease during this period presented the opportunity to investigate differences in disease-causing isolates in a relatively stable human population, thereby minimizing the confounding effects caused by population differences. The goal of this study was to identify differences that may explain clonal emergence and the cyclical nature of meningococcal disease. In addition, this study identified conserved antigens that could be investigated as potential vaccine candidates. To be responsible for the emergence of the late strain type, the differences found between the sequenced genomes of the early and late strain type must be found in the population of isolates from the time period of emergence, not just in the sequenced genomes. Therefore, we investigated target genes in isolates selected from early and late strain type populations. While some genes showed consistent allelic differences between early and late strain type population isolates, some did not. These results contribute to our understanding of which genes may be important in clonal emergence in *N. meningitidis*.

## Materials and Methods

### Selection and Characterization of Study Isolates

The study isolates were obtained from the Maryland Active Bacterial Core surveillance (ABCs) site, which conducts active, population- and laboratory-based surveillance for meningococcal infection throughout Maryland and is a component of the multistate Emerging Infections Program Network [11]. Early and late strain type ST-23 complex serogroup Y isolates were characterized by PFGE, MLST, and OMP genotyping as previously described [2], [5]. The early and late populations both contained one predominant PFGE type, so a representative isolate from each PFGE type was selected for whole genome sequencing. The date of isolation for the early strain type isolate (NM220) was June 1999 and for the late strain type isolate (NM233) was October 1999; dates of isolation were proximate by design. Both early and late profile isolates caused disease throughout the 1990s. Isolates for sequencing were chosen from the period with a peak in incidence. The proportion of disease caused by late profile isolates relative to early profile isolates was steady at this time but the incidence of disease caused by early strain type isolates was decreasing. NM220 (ST-23) and NM233 (ST-1621) are both from clonal complex 23 (ST-1621 is a single locus variant of ST-23).

To determine if gene differences between NM220 and NM233 were representative of the wider populations of disease-causing isolates, we selected 8 early strain type isolates and 8 late strain type isolates. Strains were chosen to be temporally similar between the early and late populations.

### Genome Sequencing

DNA was prepared by phenol-chloroform extraction from overnight cultures of isolated colonies grown on chocolate agar. Pyrosequencing using a GS-20 sequencer followed by assembly using the Newbler program (Roche) were accomplished by 454 Life Sciences and resulted in draft genomes. The average depth of coverage for all contigs was 22 for the early strain type and 27 for the late strain type (see Table 1). Other general features of the genomes are summarized in Table 1. A second round of pyrosequencing was accomplished using a 3kb paired end protocol. ORFs from the second sequencing were not manually curated, except for verification of selected differences. Genomes were compared between new and old sequences to identify regions missed in initial sequencing and to verify SNP differences. Missing regions were predominantly located in transposases and repeat regions.

**Table 1:** General Features of Sequenced Genomes.

Features	NM220 (early strain type)	NM233 (late strain type)
**Coverage of sequence**	21.60	27.25
**Average sequence read length**	242.869	246.036
**Number of contigs**	380	343
**Genome size (bp)**	2011600	1992570
**G+C content (%)**	51.7	51.8
**Large Contig #** 1	288	261
**Large Contig (bp)**	1992450	1977986
**Contig N50 (bp)** 2	10891	12240
**Number of predicted ORFs** 3	1896	1879
**Average gene length (bp)**	898	900
**Q40+ Bases, %** 4	99.71%	99.86%

1 Large Contig, contig at least 500 bases in length.

2 Contig N50, length such that 50% of the assembled genome lies in contigs of N50 size or longer.

3 ORF, open reading frame.

4 Q40+ Bases, all the bases in the assembled contigs that carry a phred-equivalent quality score of 40 and above. A score of 40 is equivalent to an accuracy of 99.99%.

**Table 2:** Genes found in a majority of sequenced genomes but absent from NM220 and NM233.

Protein Name	Representative Gene MC58 Genome
NadA	NMB_0394
TspB	NMB_1548
TspB	NMB_1628
TspB	NMB_1747
zonula occludens toxin family protein	NMB_1551
zonula occludens toxin family protein	NMB_1626
zonula occludens toxin family protein	NMB_1749
tellurite resistance protein TehA	NMB_1603
oxidoreductase, zinc-binding dehydrogenase family	NMB_1395
peptidase, S24 family	NMB_0910
PIN domain protein	NMB_1665
death-on-curing family protein	NMB_0917
Fic family protein	NMB_0255
DNA-cytosine methyltransferase	NMB_0725
putative plasmid toxin protein PemI	NMB_0914
putative plasmid toxin protein PemK	NMB_0913
hemagglutinin/hemolysin family protein	NMB_0493
hemagglutinin/hemolysin family protein	NMB_1214
hemolysin secretion/activation protein, ShlB family	NMB_1762
hemolysin-activating acyltransferase, HlyC family protein	NMB_1763
hemolysin-activating lysine-acyltransferase hlyC	NMB_1210
HmbR	NMB_1668

**Table 3:** Genes found in NM220 and NM233 but not in other sequenced genomes.

Protein Name	NMY220 Gene
RelA/SpoT domain protein	NMY220_01221
Exl2	NMY220_01514
hypothetical protein	NMY220_01650
ATPase RavA	NMY220_01800
putative uncharacterized protein	NMY220_01801

PCR and Sanger sequencing were used as needed for resolution of homopolymer differences and verification of relevant insertions, deletions and point mutations, using primers designed from the whole genome sequences. Primers for amplification and sequencing of gene targets in the early and late strain type populations were obtained from the literature or were designed from whole genome sequences.

### Annotation

Prediction and annotation of open reading frames (ORFs) was accomplished using a suite of automated tools combining Glimmer gene prediction [12], [13] and ORF and non-ORF feature identification (*e.g.* protein motifs) using tRNAscan-SE [14], RNAmmer [15], hmmpfam [16], [17], blastp [18], SignalP [19], prosite [20], LipoP [21], and tmhmm [22]. Automated functional annotation based on database matches was followed by automated annotation improvement through mummer-based [23] mapping from the published MC58 genome [24]. When a frameshift/point mutation was identified, the start and stop site coordinates were adjusted to encompass the entire gene. Since 454 sequencing causes frameshifts, many of the frameshifts are unlikely to be real. Therefore, where possible for the construction of orthologous clusters, the genes were translated excluding the frameshifted base(s) using a blast based algorithm implemented in Manatee [manatee.sourceforge.net]. ORFs of <50 aa in length and lacking functional evidence were removed from the genome if they were overlapping genes with functional annotation or known repeats (e.g. Correia repeats) or if they lacked an appropriate third position skew. Annotations were improved by using MugsyAnnotator [25] and manual curation using Manatee on the ortholog clusters.

### Genome Comparisons

Two methods were used to predict orthologous genes across the two serogroup Y genomes and all published *N. meningitidis* genomes [24], [26], [27], [28], [29]. First, Jaccard ortholog clusters (JOCs) were obtained from bi-directional best blastp matches [30]. JOCs are based solely on protein homology and do not utilize available information about gene synteny. Therefore, MugsyAnnotator was used to identify a second set of orthologs using whole genome alignment to better identify orthologs with conserved synteny [25]. The conservation of ortholog clusters across the various genomes analyzed was determined using Sybil, a web-based software package for comparative genomics [31], as described previously [30], [32], [33]. Since 454 draft genomes are prone to errors in homopolymers and many neisserial genes are known to be phase-variable, ORFs were not considered to be different if their amino acid sequences differed only because of a frameshift in a homopolymer or phase-variable region. With phase-variation, such differences would be characteristic of the individual isolates in culture and would not reflect differences in the population of strains. Due to the draft nature of the early and late strain type genomes, some repeat regions and genes such as transposases which occur in multiple copies within a genome were not resolved and were excluded from analysis. All well-resolved ORFs were compared between early and late strain type genomes, including, but not limited to, known antigens and potential antigens, which were defined as ORFs having lipoprotein attachment sites and/or signal sequences.

ClustalW2 [34] (http://www.ebi.ac.uk/Tools/msa/clustalw2/) was used for further investigation of nucleotide and amino acid sequence alignments. An unrooted phylogenetic network was created by aligning the genomes with Mugsy, using all of the positions present in all 22 available *N. meningitidis* genomes (1,535,077 bp) to determine the phylogenetic network with the NeighborNet algorithm using SplitsTree4 [35].

π_S_ (number of synonymous changes per synonymous site) and π_N_ (number of non-synonymous changes per non-synonymous site) for all ORFs were calculated using codeml in the PAML package [36] from within IDEA [37]. Input nucleotide alignments were generated by using the EMBOSS tranalign tool [38] on the protein alignments of the clusters as generated by Muscle [39]. Average π_S_ and π_N_ was calculated for the set of all genes, not just those with differences.

ORFs from 20 other available sequenced genomes [29] were compared to predicted ORFS of NM220 and NM233 genomes. Available genomes were grouped by clonal complex for comparison (Table S1). Absence of ORFs not meeting the previously described limits for orthologs was validated using Standalone BLAST against the nucleotide sequence of NM220 and NM233. Only ORFs not identified as orthologs and not found by Standalone BLAST analysis, with criteria of ≥80% nucleotide identity and ≥50% coverage were considered to be absent from the serogroup Y genomes.

### Nucleotide Sequence Accession Numbers

This Whole Genome Shotgun project has been deposited at DDBJ/EMBL/GenBank under the accession AGRQ00000000, AGRR00000000. The version described in this paper is the first version, AGRQ01000000, AGRR01000000. The following additional sequences for these genomes have also been deposited in GenBank: NM220 *pilC1* (JN681261), *pilC2* (JN681262), *pilE/pilS* (JN681263); NM233 *hpuA* (JN681260), *pilE/pilS* (JN681264).

## Results

### General Features of Sequenced Genomes

The two study serogroup Y genomes were similar in size and G+C content to previously sequenced neisserial genomes (Table 1). Differences in gene content between the serogroup Y genomes and the aggregate for each clonal complex ranged from 60 to 130 ORFs. The majority of gene differences were found in genes annotated as encoding hypothetical proteins, conserved hypothetical proteins, putative proteins, restriction modification systems, and phage-related proteins. Some genes were found in a majority of non-serogroup Y genomes. These include the genes *nadA*, *tspB,* and the hemoglobin receptor gene *hmbR*. Some representative genes found in a majority of other genomes but missing in the serogroup Y genomes are listed in Table 2. Both serogroup Y genomes lack the islands of horizontal transfer PNM1 (20 ORFs), IHT-C (30 ORFs) and IHT-E (19 ORFs), which contain genes encoding mainly hypothetical proteins, phage related proteins, and a transposon [24], [40]. Only five genes were found in the serogroup Y genomes but not in any other sequenced genomes (Table 3).

### Genetic Similarity between Early and Late Strain Types

**Figure 1:**
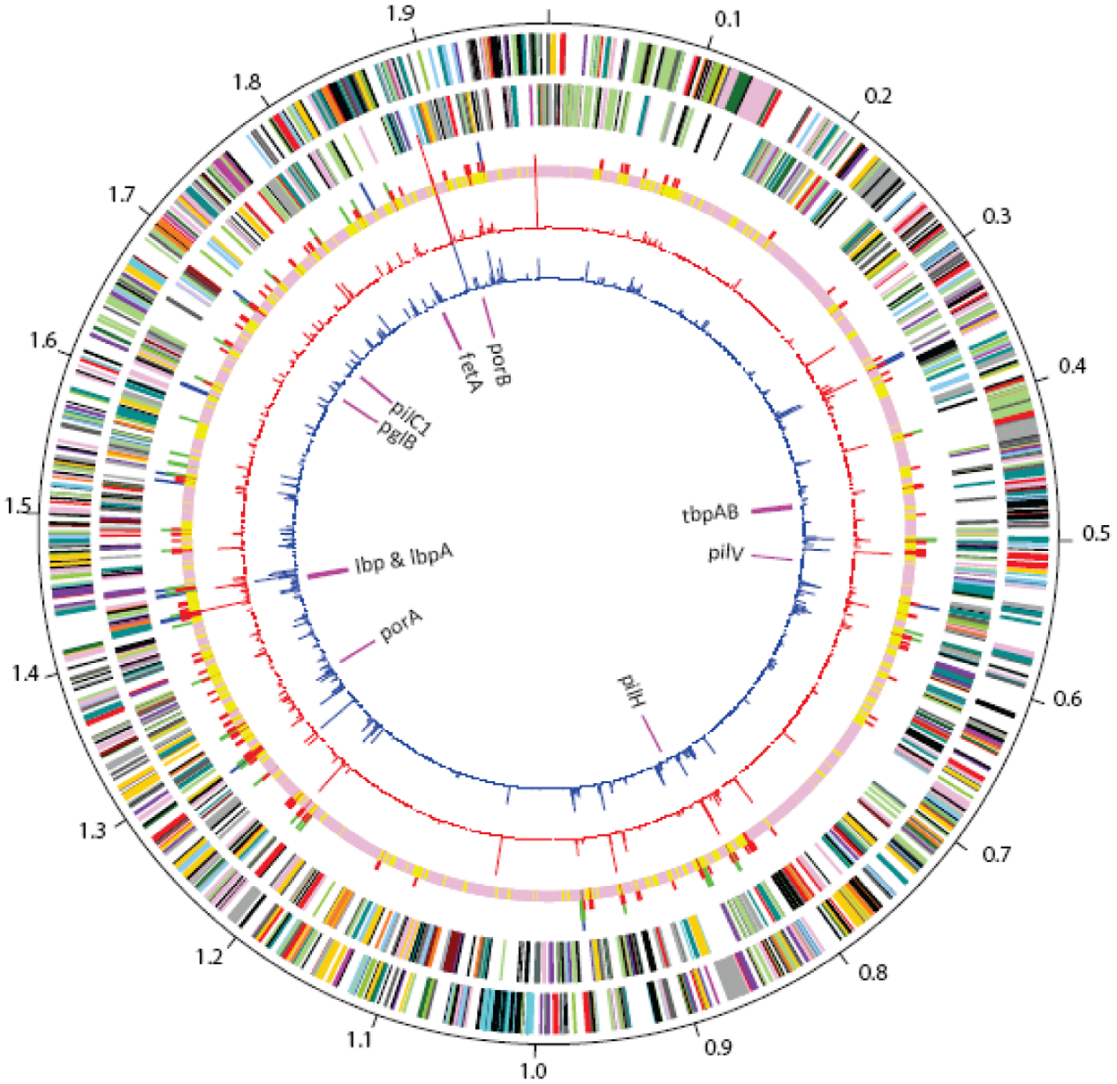
Circular representation of similarity of late strain type genome to early strain type genome. Ordered from outermost to innermost, the rims show: rim 1 (plus strand) and rim 2 (minus strand), predicted coding regions, colored by role category; rim 3, SNP density; rim 4, π_N_, plotted from 0 (base of rim) to 0.1; rim 5, π_S_, plotted 0 (base of rim) to 0.5. Role category colors are: amino acid biosynthesis, violet; biosynthesis of cofactors, prosthetic groups and carriers, light blue; cell envelope, light green; cellular processes, red; central intermediary metabolism, brown; disrupted reading frame, black; DNA metabolism, gold; energy metabolism, light gray; fatty acid and phospholipid metabolism, magenta; hypothetical proteins, black; mobile and extrachromosomal element functions, cyan; protein fate, pink; protein synthesis, pink; purines, pyrimidines, nucleosides and nucleotides, orange; regulatory functions, olive; signal transduction, olive; transcription, dark green; transport and binding proteins, blue-green; unknown function, gray; viral functions, gray. SNP density: pink, 0 SNPs per kbp; yellow, 1–25 SNPs per kbp; red, 26–50 SNPs per kbp; green, 51–75 SNPs per kbp; blue, 76–155 SNPs per kbp.

**Figure 2:**
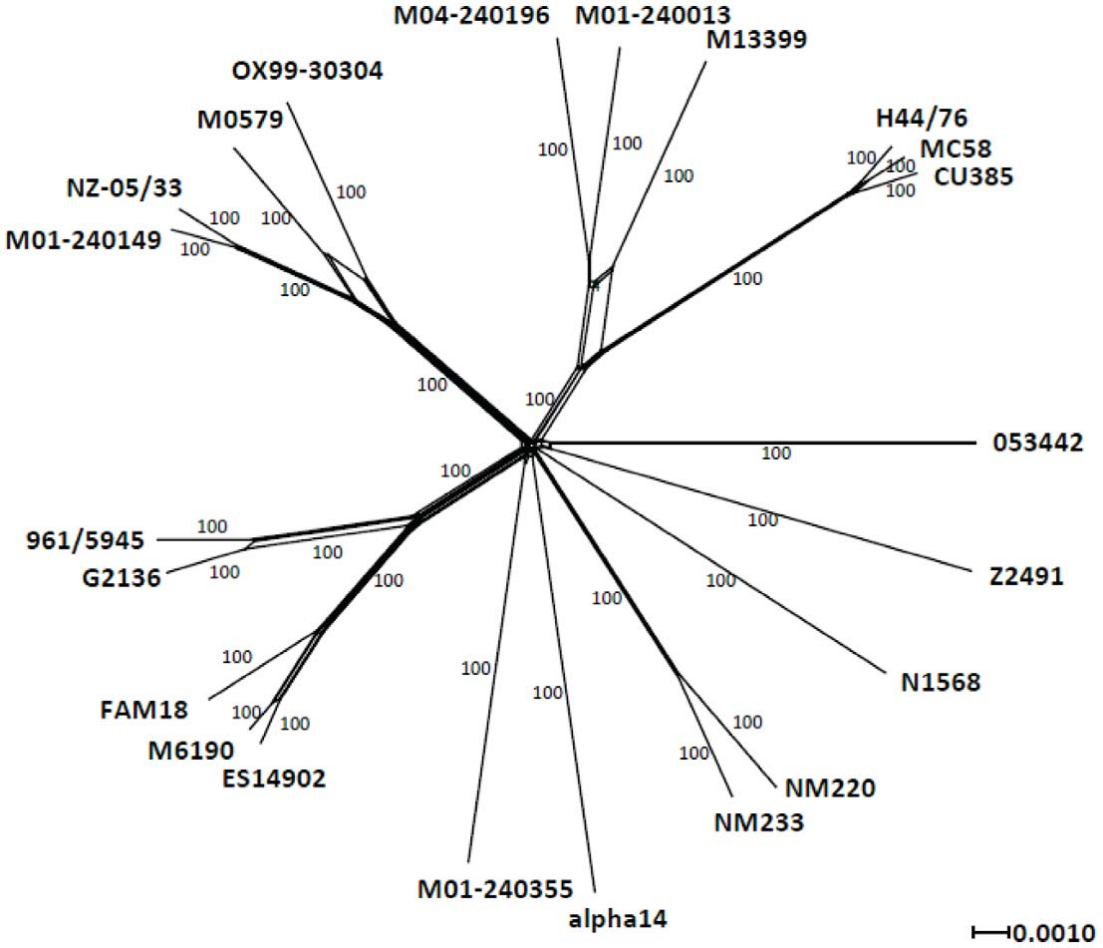
NeighborNet tree of genetic relatedness of early strain type, late strain type and 20 available sequenced meningococcal genomes using aligned sequence of regions common to all genomes. Tree produced using SplitsTree. Other genomes include: GB013, 053442, 961–5945, NZ05/33, CU385, M01-240355, M01-240149, M13399, M04-240196, H44/76, N1568, M6190, Z2491, G2136, M0579, FAM18, MC58, OX99-30304, alpha14, and ES14902.

**Figure 3:**
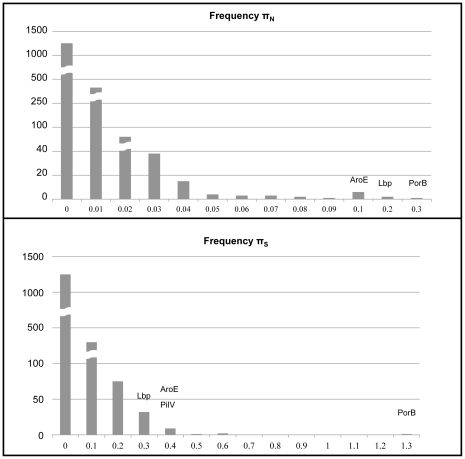
Histograms showing π_N_ (number of non-synonymous substitutions per non-synonymous site) and π_S_ (number of synonymous substitutions per synonymous site) in early strain type and late strain type genomes. π_N_ and π_S_ calculated using codeml from the PAML package.

Overall, the two genomes were highly related (Figure 1). Of 1776 shared ORFs, 1231 showed 100% amino acid identity, with a further 319 having between 99% and 100% identity. In contrast, a comparison including the genomes MC58, Z2491, 053442, FAM18, alpha14, and N1568 yielded 1490 shared ORFs, with 80 exhibiting 100% identity and an additional 538 having between 99 and 100% amino acid identity. Overall, NM220 (early strain type) and NM223 (late strain type) had 13,950 SNPs, excluding insertions and deletions that cannot be systematically examined as these are draft genomes with sequencing gaps in different locations. Repeat pyrosequencing resulted in confirmation of 10,317 SNP differences between NM220 and NM233. Not all SNPs were resolved, including regions present in one genome but missing from the other. Alignment of nucleotide sequences of the early strain type, the late strain type and a subset of other available sequenced genomes showed a high level of nucleotide conservation between the early and late strains (Figure S1). A phylogenetic network showed the early strain type and late strain type as most closely related among all currently available sequenced meningococcal genomes (Figure 2), with the serogroup Y genomes clustering in a branch not closely related to other genomes. Values for π_N_ and π_S_ were low over a majority of the genomes (Figures 1 and 3) with average π_N_ = 0.0033 and average π_S_ = 0.0216. No non-synonymous changes were found in 1238 shared ORFs and no synonymous changes were found in 1195 shared ORFs. ORFs with the highest π_N_ and π_S_ included antigens, predicted membrane proteins, hypothetical proteins and housekeeping genes (Table 4).

**Table 4:** Genes with highest π_S_ and π_N_ in the early strain type and late strain type genome comparison. Maximum likelihood π_S and_ π_N_ calculated using the codeml program from PAML.

Highest π_S_					Highest Π_N_				
π_N_	π_S_	Early	Late	Predicted Protein	π_N_	π_S_	Early	Late	Predicted Protein
0.2789	1.2413	NMY220_1828	NMY233_1807	PorB	0.2789	1.2413	NMY220_1828	NMY233_1807	PorB
0.0372	0.5443	NMY220_1280	NMY233_1266	glucokinase	0.1793	0.2512	NMY220_1916	NMY233_1893	conserved hypothetical protein
0.0786	0.5293	NMY220_1431	NMY233_1409	putative permease, YjgP/YjgQ family	0.1559	0.2387	NMY220_1413	NMY233_1391	lactoferrin-binding protein
0.0406	0.4260	NMY220_1858	NMY233_1837	thiamine biosynthesis protein ThiS	0.0980	0.0821	NMY220_0860	NMY233_0849	prepilin-type N-terminal cleavage/methylation domain protein
0.0173	0.3737	NMY220_0538	NMY233_0511	magnesium and cobalt efflux protein CorC	0.0964	0.1445	NMY220_0962	NMY233_0948	conserved hypothetical protein
0.0375	0.3675	NMY220_1299	NMY233_1283	conserved hypothetical protein	0.0959	0.2951	NMY220_1241	NMY233_1228	conserved hypothetical protein
0.0936	0.3545	NMY220_0390	NMY233_0372	shikimate 5-dehydrogenase (AroE)	0.0951	0.2212	NMY220_1068	NMY233_1071	putative lipoprotein
0.0299	0.3518	NMY220_1433	NMY233_1411	ornithine carbamoyltransferase	0.0936	0.3545	NMY220_0390	NMY233_0372	shikimate 5-dehydrogenase (AroE)
0.0266	0.3293	NMY220_0388	NMY233_0370	lipopolysaccharide ABC transporter,ATP-binding protein	0.0921	0.3260	NMY220_0548	NMY233_0521	PilV
0.0921	0.3260	NMY220_0548	NMY233_0521	PilV	0.0786	0.5293	NMY220_1431	NMY233_1409	putative permease, YjgP/YjgQ family
0.0256	0.3244	NMY220_0961	NMY233_0947	phosphoribosyl aminoimidazolecarboxylase, ATPase subunit	0.0754	0.2449	NMY220_0822	NMY233_0800	conserved hypothetical protein
0.0334	0.3023	NMY220_1434	NMY233_1412	ketol-acid reductoisomerase	0.0628	0.1951	NMY220_1465	NMY233_1442	two component sensor kinase
0.0239	0.3011	NMY220_1869	NMY233_1848	cysteinyl-tRNA synthetase	0.0613	0.2013	NMY220_0445	NMY233_0426	sel1 repeat protein
0.0959	0.2951	NMY220_1241	NMY233_1228	conserved hypothetical protein	0.061	0.2852	NMY220_0858	NMY233_0847	prepilin-type N-terminal cleavage/methylation domain protein
0.0107	0.2890	NMY220_0977	NMY233_0981	conserved hypothetical protein	0.0544	0.2142	NMY220_0600	NMY233_0574	septum formation protein Maf
0.0610	0.2852	NMY220_0858	NMY233_0847	prepilin-type N-terminal cleavage/methylation domain protein	0.0544	0.2142	NMY220_0977	NMY233_0981	conserved hypothetical protein

### Vaccine Candidates and Conserved Antigens

Three proteins, factor H binding protein (FHbp), neisserial heparin binding antigen (NhbA), and NadA have been investigated as targets for vaccines against serogroup B disease [41], [42]. The genes encoding FHbp are identical in the early and late strain types (Table S2) while the genes for NhbA exhibited 99.8% predicted amino acid identity, with only 2 predicted amino acid changes (Table S3). In both genomes the gene encoding the protein product NadA is missing and that section of the genome contains the sequence 5′-TTTCCATTCCAAACGC-3′. This situation has been described in other NadA deficient strains [43]. Lipoprotein prediction based on sequence identified a total of 73 shared lipoprotein ORFs in the early and late strain types. Forty-three of the shared predicted lipoprotein ORFs had 100% nucleotide and amino acid identity between the early and late strain types (Table S2) and three others had only synonymous nucleotide changes (Table S3). Comparison of the amino acid identity of the conserved lipoproteins with 100% nucleotide identity, across the twenty additional genomes which have been completely sequenced, showed that the majority were highly conserved across those genomes. These highly conserved lipoproteins may be of interest as further vaccine candidates should they be found to elicit an appropriate immune response.

### Antigenic Differences

The gene encoding the antigenic outer membrane protein PorB had the highest π_N_ and π_S_ in the early strain type/late strain type comparison (π_N_ = 0.2789, π_S_ = 1.2413). Other antigens with relatively high π_N_ and/or π_S_ included lactoferrin binding protein B (LbpB) and PilV (Table 4).

### PorA, PorB, FetA

In a previous study, we demonstrated differences in deduced PorA variable regions (VR1 and VR2), PorB loops V and VII, and FetA VR [2]. Comparison of entire sequences of these proteins showed additional changes. The predicted amino acid sequence of late strain type PorA contained 6 amino acid changes relative to the early strain type sequence, in addition to those previously reported. PorB sequences exhibited more extensive differences, with the predicted amino acid sequence containing 5 amino acid insertions and 93 amino acid substitutions in addition to those previously reported in loops V and VII (Figure S2). Late strain type FetA contained 1 amino acid deletion and 20 amino acid substitutions in addition to those previously reported in the variable region [2].

### Lipoproteins and Proteins with Signal Sequences

The early and late strain type genomes contained 456 shared ORFs which are potential antigens based upon predicted lipoprotein attachment sites and/or predicted signal sequences. Of 73 shared lipoproteins, 27 exhibited amino acid differences (Table S3). Of the 383 ORFs with signal sequences but no lipoprotein attachment sites, only 68 had ≤99% amino acid identity. The majority of these ORFs were associated with the cell envelope (22) or with transport and binding (17), based on their gene role category. Differences were found in the iron acquisition and uptake lipoproteins LbpB (Figure S3) and hemoglobin-haptoglobin utilization protein A (HpuA) (Figure S4). The putative lipoprotein and suggested vaccine target Ag473 [44] contains a 21 nt tandem repeat with a 2 repeat difference between early and late strain type, resulting in a 14 aa insertion in the late strain type predicted protein. Differences were also noted in the non-lipoprotein iron acquisition and uptake proteins transferrin binding protein A (TbpA), transferrin binding protein B (TbpB), and lactoferrin binding protein A (LbpA) (Table S3).

### Pilus Structure and Assembly

*N. meningitidis* has type IV pili **(**Tfp**)** which are polymers whose major subunit is the protein product of the highly variable *pilE* gene [45], [46]. Due to the highly repetitive nature of this section of the genomes, initial sequences were fragmentary. Further 454 sequencing of this region indicated that the *pilS* regions of the early strain type and the late strain type were nearly identical over part of the sequence. However, the early strain type contained fewer *pilS* cassettes (3 cassettes, the late strain type contains 5 cassettes) and the *pilS* region of the late strain type contained a putative IS*1160*-like transposase between the fourth and fifth *pilS* cassettes (GenBank accession numbers JN681263, JN681264). The sequence of the *pilE* gene was similar between the early and late strain types over the C-terminal half but highly dissimilar at the N-terminus. The late strain type transposase was only partially resolved and further sequencing was not undertaken.

The predicted pilus adhesin, PilC1 (GenBank accession number JN681261and NMY233_1622) exhibited 84% amino acid similarity between the early strain type and the late strain type (Figure S5). Minor pilin PilV is encoded in the early and late population strains by the genes NMY220_0548 and NMY233_0521, respectively. The predicted PilV amino acid identity between the early strain type and the late strain type is 91.5%, with differences in 22 of the total 130 amino acids. The genes bounding the *pilV* locus have ≥ 98% nucleotide identity and ≥ 99% amino acid identity between the two strain types, indicating that this gene may have participated in horizontal gene transfer.

The early strain type contained the PglB form of the pilin glycosylation B locus (NMY220_1618) while the late strain type contained PglB2 (NMY233_1594). In the late strain type, PglB2 was immediately followed by a conserved hypothetical protein (NMY233_1595) of unknown function [47] not found in the early strain type.

### Genes Found Exclusively in Early or Late Strain Type

The early strain type and late strain type each contained genes not found in the other genome (Table S4). The majority of these genes encoded hypothetical proteins, putative lipoproteins or putative membrane proteins. The late strain type contained two genes encoding proteins with significant similarity to proteins with experimentally verified functions: *zitB* (NMY233_0596), which encodes a predicted cation-efflux facilitator family protein, and a gene encoding a rubredoxin (NMY233_0921).

### Serogroup Y Clonal Complex 23 Population Results

To determine whether the differences identified in the single sequenced early and late strain type isolates were characteristic of the larger population of isolates, ten gene targets were investigated in eight early strain type and eight late strain type population isolates. The early strain type and late strain type population isolates were consistent with NM220 and NM233 in gene content for *pglB* and *pglB2*, *zitB*, and rubredoxin (Table 5, presence or absence by PCR). The sequenced alleles of *hpuA* in the population strains were also identical to the NM220 and NM233 alleles by strain type (all early strain type matched NM220, all late strain type matched NM233), as were the alleles for *pilV*. Tandem repeat differences in *ag473* were maintained in population isolates, except for one early strain type isolate that had a single tandem repeat, and thus did not match either the early or late strain type. Late strain type population *lbpB* alleles were identical to the NM233 allele. Among the early strain type isolates, five *lbpB* alleles were identical to the NM220 allele but NM109, NM115 and NM206 had an allele that differed from both NM220 and NM233. All late strain type *tbpB* alleles were identical. Early strain type isolates contained three alleles for *tbpB*, all of which differed from the alleles found in either NM220 or NM233, and two isolates failed to amplify that locus.

**Table 5:** Early and late strain type population isolates, year of isolation, and results of PCR and Sanger sequencing of target genes.

ID	Year	Strain Type^1^	Gene Targets										
			*pglB/pglB2*	*zitB*	*rubredoxin*	*hpuA*	*lbpB*	*tbpB*	*pilV*	*pilH*	*pilI*	*pilJ*	*ag473*
NM 109	1996	Early	*pglB*	NP^2^	NP	E^3^	L^4^	V^15^	E	L	L	L	E
NM 115	1996	Early	*pglB*	NP	NP	E	L	V1	E	L	L	L	E
NM 131	1996	Early	*pglB*	NP	NP	E	E	V1	E	L	L	L	E
NM 187	1998	Early	*pglB*	NP	NP	E	E	F^6^	E	E	E	E	E
NM 206	1999	Early	*pglB*	NP	NP	E	L	V1	E	L	L	L	E
NM 235	1999	Early	*pglB*	NP	NP	E	E	F	E	L	L	L	E
NM 271	2001	Early	*pglB*	NP	NP	E	E	V^27^	E	L	L	L	R^8^
NM 284	2001	Early	*pglB*	NP	NP	E	E	M^9^	E	L	L	L	E
NM 289	2001	Early	*pglB*	NP	NP	E	E	V2	E	E	E	E	E
NM51	1993	Late	*pglB2*	P^10^	P	L	L	L	L	L	L	L	L
NM101	1995	Late	*pglB2*	P	P	L	L	L	L	L	L	L	L
NM119	1996	Late	*pglB2*	P	P	L	L	L	L	L	L	L	L
NM145	1997	Late	*pglB2*	P	P	L	L	L	L	L	L	L	L
NM165	1997	Late	*pglB2*	P	P	L	L	L	L	L	L	L	L
NM203	1999	Late	*pglB2*	P	P	L	L	L	L	L	L	L	L
NM249	2000	Late	*pglB2*	P	P	L	L	L	L	L	L	L	L
NM261	2000	Late	*pglB2*	P	P	L	L	L	L	L	L	L	L
NM264	2000	Late	*pglB2*	P	P	L	L	L	L	L	L	L	L

1 Based upon OMP and PFGE profile [2];

2 NP, not present, by PCR;

3 E, early strain type allele;

4 L, late strain type allele;

5 V1, *tbpB* variant allele 1;

6 F, failed PCR;

7 V2, *tbpB* variant allele 2;

8 R, repeat difference;

9 M, mosaic allele;

10 P, present, by PCR.

NM220 and NM233 contained different alleles for three pilin-associated genes *pilH*, *pilI*, and *pilJ*. The population of early strain type isolates contained the late strain type allele except for 2 isolates (isolated in 1998 and 2001), which contained the early strain type alleles.

## Discussion

Some studies have compared whole neisserial genomes in an attempt to elucidate the basis for differences in invasiveness and pathogenicity [26], [48]. Other than the presence of a capsule and the production of endotoxin, no definitive requirements for virulence and pathogenicity have been found. All sequenced genomes differ in gene content from each other; the absolute number of differences is understandably greater in strains from different clonal complexes. Even within a clonal complex, some genes are present in some strains but not in all. Some of these variably present genes are phage-related and their presence/absence may reflect evolutionary distance since the time of phage acquisition. Many other variably present genes are annotated as encoding hypothetical proteins, with insufficient similarity to well-characterized genes to allow prediction of function.

A phylogenetic tree of genetic relatedness of early strain type, late strain type and 20 available sequenced meningococcal genomes indicates that the serogroup Y strains are most closely related to each other and they cluster in a branch not closely related to other sequenced genomes. The serogroup Y strains in this study differ in gene content from other sequenced genomes in the same way as other sequenced genomes differ from each other, that is, mainly in phage-related genes and in gene encoding hypothetical proteins. Most notably, the serogroup Y genomes are missing the genes located in the putative islands of horizontal transfer PNM1, IHT-C and IHT-E, all of which appear to be of phage origin. It has been suggested that these genes contribute to virulence and pathogenicity, but their actual roles are currently unknown [40], [49].

Closely related strains that differ in their ability to cause disease are ideal for investigating the genetic factors that contribute to that ability to cause disease [50]. An analogous situation is presented in the case of replacement of one invasive strain by another over time, where the analysis involves determination of factors responsible for the decrease in incidence of the earlier strain and increase in incidence of the later strain. In this study, we used pyrosequencing to obtain draft genomes of two closely related clonal complex 23 meningococcal strains from Maryland: one whose ST, OMP profile and PFGE profile were representative of a strain causing disease in the early 1990s and one with a different OMP and PFGE profile which was responsible for disease later in that decade [2]. The early and late strains exhibited antigenic differences which can be postulated to have contributed to the decline of the early strain type and the emergence of the late strain type.

The majority of ORFs in the two genomes had few or no mutations, leading to an overall low level of π_N_ and π_S._ The highest value of both π_N_ and π_S_ was found in the gene encoding PorB, which is known to be both immunogenic and highly variable. Other genes with relatively high π_N_ and π_S_ included those encoding PorA, FetA, PilV, LbpB, and HpuA. A number of housekeeping genes were represented among those with high π_N_ and π_S_, including shikimate 5-dehydrogenase (*aroE*), one of the seven genes used for MLST. The late strain type is a single locus variant of ST-23; single locus variants are considered to have arisen by horizontal gene transfer if the variant allele exhibits extensive differences from the allele found in the parent sequence type. Therefore, the π_N_ and π_S_ values for shikimate 5-dehydrogenase can be used as a marker for horizontal gene transfer. Genes in the early and late genomes whose π_N_ and π_S_ are similar to that of *aroE* have likely been involved in horizontal gene transfer.

In addition to the overall low values of π_N_ and π_S_, loci which differed between the early and late strain type genomes exhibited a surplus of π_S_ over π_N_. This phenomenon has been documented in methicillin-resistant *Staphylococcus aureus* and *Clostridium difficile* [51]. Recent mutations show an excess of non-synonymous changes, which will be modified over time by the effects of selection. Alleles which result from horizontal gene transfer have a relative surplus of synonymous changes because in them, selection has already purged deleterious non-synonymous changes. The relative abundance of synonymous over non-synonymous changes in genes which differ between the early and late strains is another indication that these genes have been involved in horizontal gene transfer.

Given that both strain types caused similar rates of disease in the same population during different periods of the same decade, we did not expect major differences in virulence between them. Accordingly, our principal hypothesis was that emergence of the late strain type was primarily due to antigenic changes that allowed escape from population immunity. A number of proteins are known to be immunogenic in *N. meningitidis* and many others can be predicted to be immunogenic based upon their structure or their function. Our comparison of whole genomes found a preponderance of antigens unchanged but also found differences in a number of loci that contribute to the antigenic profile and these differences may have been instrumental in disease emergence. Genes exhibiting differences include antigenic outer membrane proteins, genes involved in pilus structure, function and glycosylation, and genes involved in iron acquisition and uptake. Differences in the genes encoding the antigens PorA, PorB, and FetA are more extensive than previously described [2]. Additional antigens differing between the early and late strain types included putative lipoproteins and the vaccine target Ag473. Neisserial type IV pili **(**Tfp**)** are necessary for adhesion of bacterial cells to human mucosal cells and are major antigens [52]. Differences were found in genes that can be expected to affect the Tfp antigen profile, including the major structural subunit PilE and in the pilus tip adhesin PilC [53]. The *pilH/I/J* loci differed between early and late strains but most strains within the population tested contained the late strain type alleles. These loci may not have been involved in emergence of the late strain type. Alternatively, the late strain type alleles may have been acquired early in the genetic transformation of the early to late strain type. The presence of the early strain type alleles in isolates obtained in three different years (1998, 1999, and 2001) may imply persistence of a sub-population possessing those alleles or multiple instances of horizontal transfer. Pilin glycosylation is believed to be an important mechanism for decreasing immunogenicity of the pilin subunits by masking exposed areas of the protein. In *N. meningitidis*, the gene encoding pilin glycosylation protein B (PglB) has been documented to exist in 2 forms, *pglB* and *pglB2* [47], [54], [55]. The early strain type contains *pglB*, while the late strain type contains *pglB2*. Based upon the number and extent of differences in genes affecting the structure and function of the pilus, it can be theorized that these differences contributed to the change in disease epidemiology.

Iron acquisition and uptake are necessary for survival of *N. meningitidis* in the human host and iron is sequestered in a variety of forms, including lactoferrin, transferrin, and hemoglobin/haptoglobin. *N. meningitidis* has evolved mechanisms for obtaining iron from these sources using several two component systems. These systems are antigenic, as they require surface exposure to bind their substrates. In addition, they are highly variable, with different alleles found in otherwise similar strains during epidemics, invoking the concept of a “genocloud”, a combination of a dominant strain and its close relatives [56]. The early and late strain type differed at genes encoding the main iron acquisition systems for lactoferrin, transferrin, and hemoglobin/haptoglobin. The alleles for these genes were consistent in a population of late strain type isolates. However, some early strain type isolates contained the late strain type allele for *lbpB* and the early strain type isolates contained multiple variants of *tbpB*, which did not match either the early or late allele. These results may indicate selection for recombinants at these loci in a population of isolates against which human immunity has developed.

We also identified genes encoding known and predicted lipoproteins that were highly similar or identical in the two genomes. Factor H binding protein (*fHbp*), neisserial heparin binding antigen (*nhbA*) and *nadA* have been identified as encoding possible targets for vaccines for prevention of serogroup B meningococcal disease as well as that caused by other serogroups [41], [42]. The gene *nadA* was absent in the early and late strain type genomes, but *fHbp* and *nhbA* were highly conserved. This study found 46 other genes encoding predicted lipoproteins that were identical in amino acid sequence between the early and late strain types and an additional 20 with greater than 99% amino acid identity. The majority of the identical lipoproteins were also highly conserved across 20 sequenced genomes, suggesting that some of them may be appropriate for investigation as vaccine targets. Conservation of antigens may indicate that structural constraints based upon function limit variation in those proteins. Conserved antigens are superior vaccine candidates, since highly variable vaccine targets complicate vaccine formulation, requiring the inclusion of many variants. However, highly conserved antigens may be a disadvantage to the bacteria, since their conservation predicts that any immunity they engender will be broad and will therefore limit spread of many strains.

Only a few genes were found in one of the genomes sequenced in this study but not the other. The majority of these were hypothetical proteins, but some are predicted to be membrane associated and therefore possibly antigenic. The exact function and importance of these genes will require further investigation.

Sequencing of a single genome provides a complete picture of that particular genome but does not provide information on variations among a population of closely related strains. While some studies have sequenced multiple strains, the relationship of a sequenced genome to a wider population of similar strains is still relatively unexplored. Our results indicate that some genes differ even within isolates from a closely related population. The identity of these genes gives important insights into the ways in which *N. meningitidis* adapts to the immune response. Allelic variation of known antigens has been demonstrated by targeted Sanger sequencing, but this method requires prior knowledge of the targeted genes. The whole genome approach allows investigation of known targets but also facilitates identification of novel genes whose importance and even function has not previously been determined.

The existence of two strains that are close temporally and geographically, with a majority of genes identical but still some genes highly variant, accentuates the mosaic nature of the meningococcal genome and the ability of this bacterium to acquire new gene variants through horizontal gene transfer and to continue to cause invasive disease. The results of this study suggest that specific genes, mostly encoding antigens, were associated with and were potentially responsible for the expansion of serogroup Y disease. However, this study represents an ecological analysis which makes causality difficult to prove. Additional studies involving multiple examples of clonal emergence of *N. meningitidis* are needed to determine whether there are consistent antigenic features associated with meningococcal disease emergence.

## Supporting Information

Figure S1 **Percent identity plots from Mugsy alignments of early strain type, late strain type, MC58, Z2491, 053442, FAM18, and alpha14 visualized using the GMAJ alignment viewer, with the early strain type genome as the reference.** The horizontal axis shows nucleotide positions in the sequence. The vertical axis shows percentage of matching nucleotides at each position. Percent identity ranges from 50 (bottom) to 100 (top) percent in each row.
(TIF)

Figure S2 **Alignment of predicted amino acid sequence of early strain type (upper sequence) and late strain type (lower sequence) PorB (NMY220_1828 and NMY233_1807) showing non-conserved amino acids.** Boxes outline loops V (*) and VII (+), which were previously reported.
(TIF)

Figure S3 **Alignment of predicted protein lactoferrin binding protein B (LbpB) showing non-conserved amino acids.** Upper sequence early clone (NM220), lower sequence late strain type (NM233).
(TIF)

Figure S4 **Alignment of predicted protein hemoglobin-haptoglobin utilization protein A (hpuA) showing non-conserved amino acids.** Upper sequence early strain type (NM220), lower sequence late strain type (NM233).
(TIF)

Figure S5 **Alignment of predicted protein PilC1, showing non-conserved amino acids.** Upper sequence early strain type (NM220), lower sequence late strain type (NM233).
(TIF)

Table S1 **Available sequenced genomes.**
(DOCX)

Table S2 **Lipoproteins exhibiting identical nucleotide and amino acid sequence in early and late clones and their amino acid identity across sequenced genomes.**
(DOCX)

Table S3 **Early clone (NM220) and late clone (NM233) differences in predicted outer membrane proteins, lipoproteins, proteins involved in pilus biogenesis and non-lipoproteins involved in iron acquisition and uptake.**
(DOCX)

Table S4 **Proteins encoded by genes found in only one of the clones.**
(DOCX)
