# Supplementary figures and images for: Whole Genome Sequencing to Investigate the Emergence of Clonal Complex 23 Neisseria meningitidis Serogroup Y Disease in the United States
Source: PLoS One. 2012 Apr 27;7(4):e35699. doi: 10.1371/journal.pone.0035699 (PMC3338715; doi:10.1371/journal.pone.0035699)

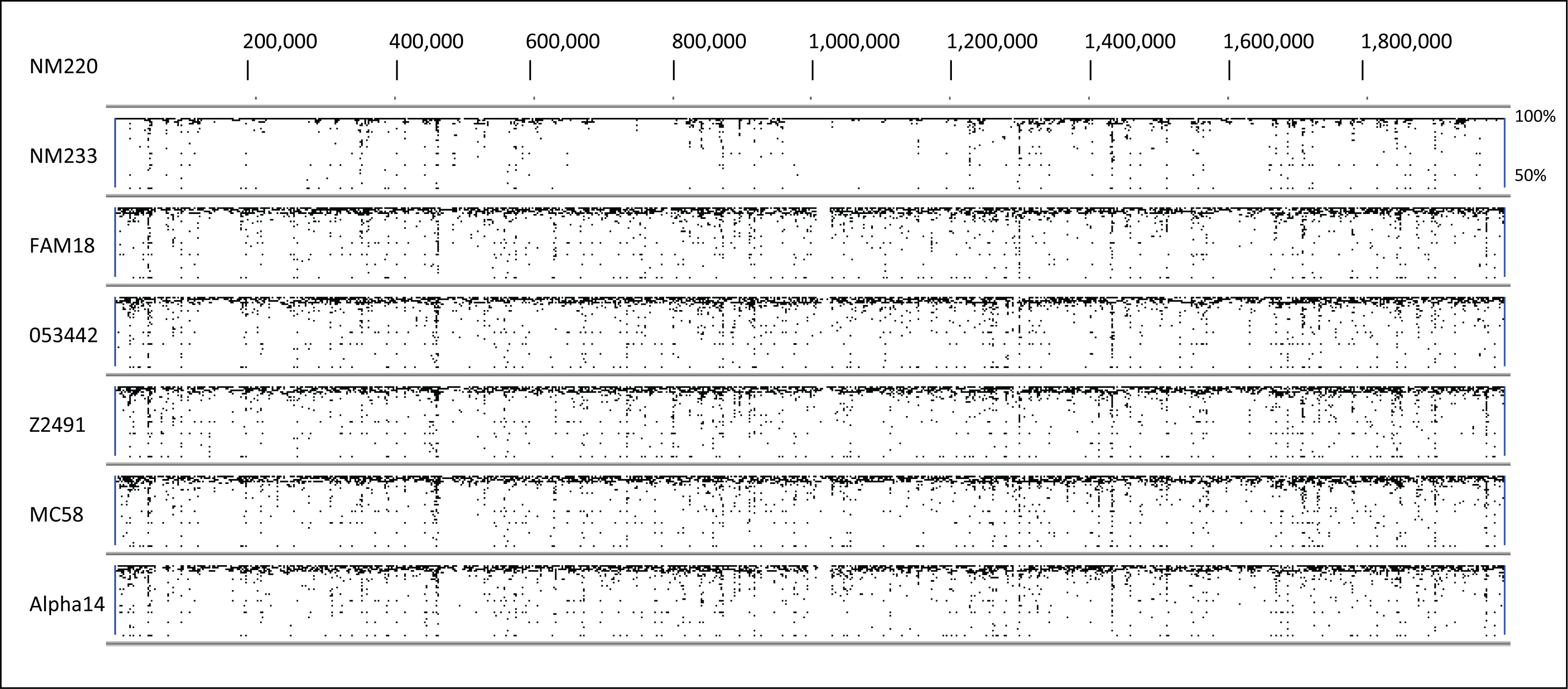

Supplement: Figure S1 — Percent identity plots from Mugsy alignments of early strain type, late strain type, MC58, Z2491, 053442, FAM18, and alpha14 visualized using the GMAJ alignment viewer, with the early strain type genome as the reference. The horizontal axis shows nucleotide positions in the sequence. The vertical axis shows percentage of matching nucleotides at each position. Percent identity ranges from 50 (bottom) to 100 (top) percent in each row. (TIF) [file pone.0035699.s001.tif]

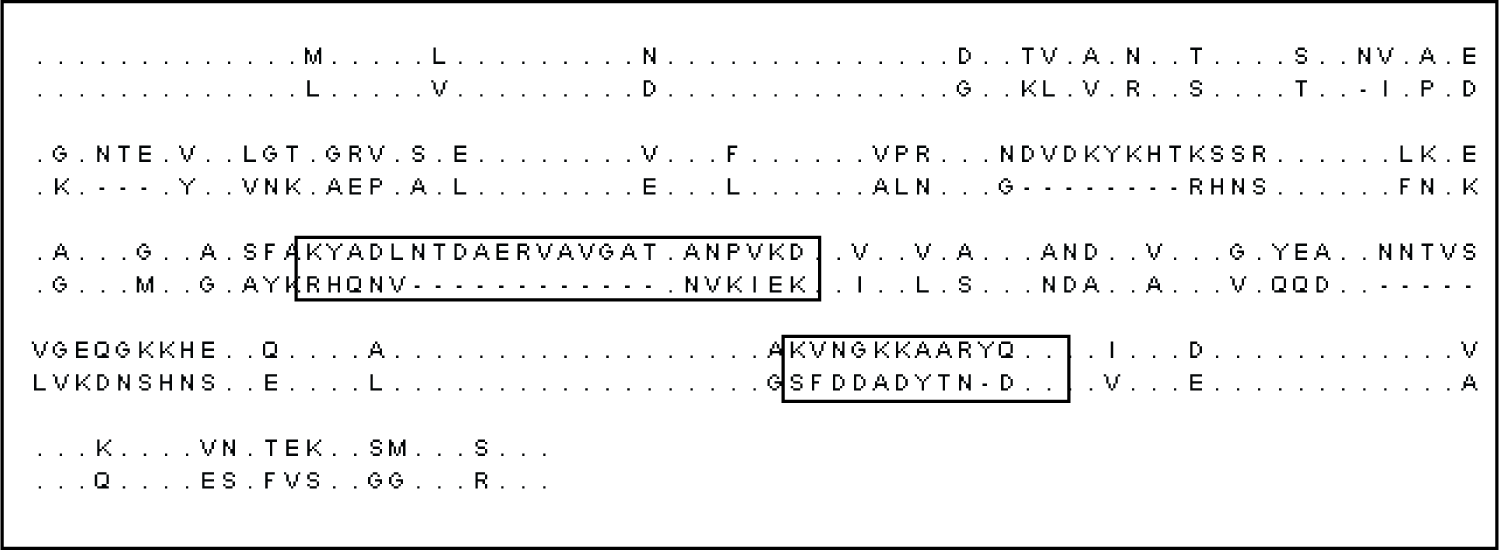

Supplement: Figure S2 — Alignment of predicted amino acid sequence of early strain type (upper sequence) and late strain type (lower sequence) PorB (NMY220_1828 and NMY233_1807) showing non-conserved amino acids. Boxes outline loops V (*) and VII (+), which were previously reported. (TIF) [file pone.0035699.s002.tif]

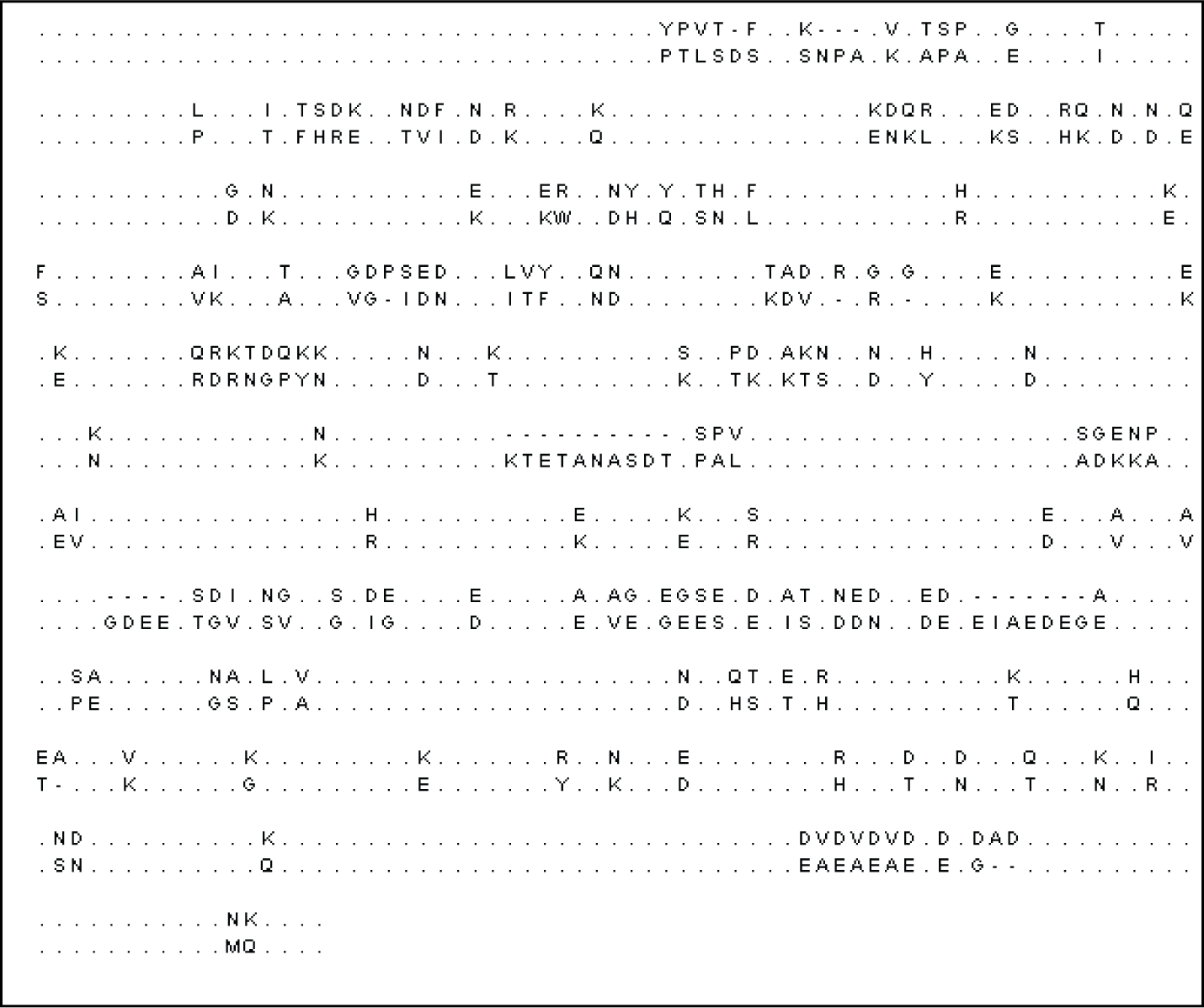

Supplement: Figure S3 — Alignment of predicted protein lactoferrin binding protein B (LbpB) showing non-conserved amino acids. Upper sequence early clone (NM220), lower sequence late strain type (NM233). (TIF) [file pone.0035699.s003.tif]

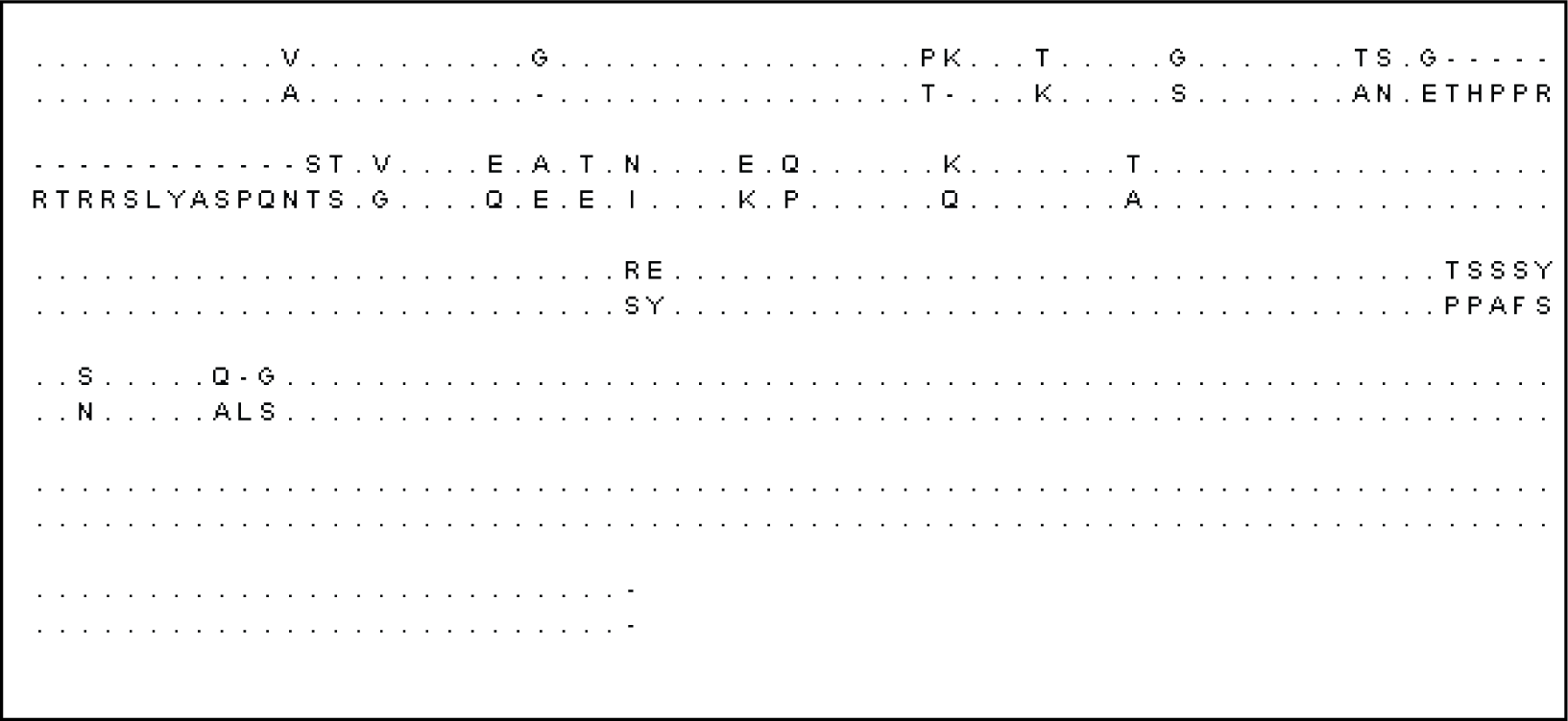

Supplement: Figure S4 — Alignment of predicted protein hemoglobin-haptoglobin utilization protein A (hpuA) showing non-conserved amino acids. Upper sequence early strain type (NM220), lower sequence late strain type (NM233). (TIF) [file pone.0035699.s004.tif]

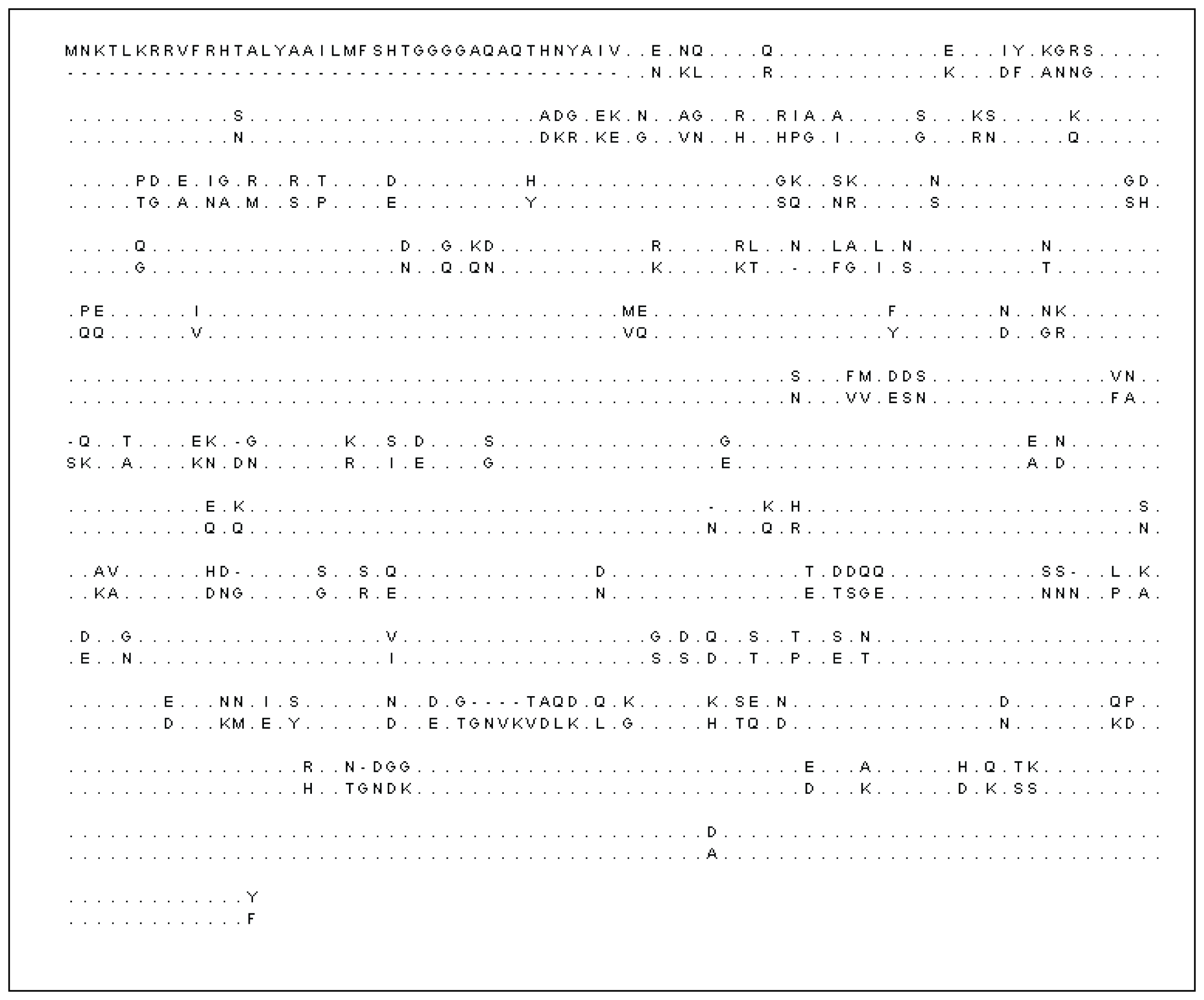

Supplement: Figure S5 — Alignment of predicted protein PilC1, showing non-conserved amino acids. Upper sequence early strain type (NM220), lower sequence late strain type (NM233). (TIF) [file pone.0035699.s005.tif]
