# Supplementary material for: Whole Genome Sequencing to Investigate the Emergence of Clonal Complex 23 Neisseria meningitidis Serogroup Y Disease in the United States
Source: PLoS One. 2012 Apr 27;7(4):e35699. doi: 10.1371/journal.pone.0035699 (PMC3338715; doi:10.1371/journal.pone.0035699)
Supplement: Table S1 — Available sequenced genomes. (DOCX) [file pone.0035699.s006.docx]

Table S1. Available sequenced genomes.

| Strain | NCBI locus tag | Serogroup | Sequence Type | Clonal Complex |
| --- | --- | --- | --- | --- |
| alpha14 | NMO | cnl | 53 | 53 |
| N1568 | NMXN1568 | X | 751 | none |
| Z2491 | NMA | A | 4 | 4 |
| 053442 | NMCC | C | 4821 | 4821 |
| FAM18 | NMC | C | 11 | 11 |
| ES14902 | NMBES14902 | B | 11 | 11 |
| M6190 | NMBM6190 | B | 1988 | 11 |
| M01-240355 | NMBM01240355 | B | 213 | 213 |
| NZ-05/33 | NMBNZ0533 | B | 42 | 41/44 |
| M0579 | NMBM0579 | B | 43 | 41/44 |
| M01-240149 | NMBM01240149 | B | 41 | 41/44 |
| OX99-30304 | NMBOX9930304 | B | 44 | 41/44 |
| M04-240196 | NMBM04240196 | B | 269 | 269 |
| M13399 | NMBM13399 | B | 2976 | 269 |
| GB013 (M01-240013) | NMBM01240013 | B | 275 | 269 |
| H44/76 | NMBH4476 | B | 32 | 32 |
| CU385 | NMBCU385 | B | 33 | 32 |
| MC58 | NMB | B | 74 | 32 |
| 961–5945 | NMB9615945 | B | 153 | 8 |
| G2136 | NMBG2136 | B | 8 | 8 |
