# Supplementary material for: Whole Genome Sequencing to Investigate the Emergence of Clonal Complex 23 Neisseria meningitidis Serogroup Y Disease in the United States
Source: PLoS One. 2012 Apr 27;7(4):e35699. doi: 10.1371/journal.pone.0035699 (PMC3338715; doi:10.1371/journal.pone.0035699)
Supplement: Table S2 — Lipoproteins exhibiting identical nucleotide and amino acid sequence in early and late clones and their amino acid identity across sequenced genomes. (DOCX) [file pone.0035699.s007.docx]

Table S2. Lipoproteins exhibiting identical nucleotide and amino acid sequence in early and late strain types and their amino acid identity across sequenced genomes.

| Early Clone | Late Clone | Protein | % Identity^1^ | Number of Genomes^2^ |
| --- | --- | --- | --- | --- |
| NMY220_0089 | NMY233_0073 | putative lipoprotein | 96.5 | 19 |
| NMY220_0090 | NMY233_0074 | mltA family protein | 99.2 | 19 |
| NMY220_0092 | NMY233_0076 | putative lipoprotein | 99.6 | 20 |
| NMY220_0108 | NMY233_0090 | putative lipoprotein | 97.5 | 20 |
| NMY220_0116 | NMY233_0098 | capsule polysaccharide export outer membrane protein CtrA | 99.3 | 20 |
| NMY220_0148 | NMY233_0130 | putative lipoprotein | 97.7 | 19 |
| NMY220_0244 | NMY233_0226 | lipoprotein, SmpA/OmlA family | 95.0 | 20 |
| NMY220_0317 | NMY233_0298 | putative thiol:disulfide interchange protein DsbA | 99.3 | 20 |
| NMY220_0334 | NMY233_0316 | DSBA thioredoxin domain protein | 99.1 | 20 |
| NMY220_0533 | NMY233_0506 | protease Do | 98.4 | 20 |
| NMY220_0581 | NMY233_0556 | nosL protein | 99.5 | 20 |
| NMY220_0749 | NMY233_0724 | putative lipoprotein | 97.4 | 20 |
| NMY220_0775 | NMY233_0750 | L-cystine ABC transporter, periplasmic L-cystine-binding protein | 98.6 | 20 |
| NMY220_0870 | NMY233_0859 | cytochrome c, class II | 97.0 | 19 |
| NMY220_0938 | NMY233_0924 | putative lipoprotein | 99.8 | 20 |
| NMY220_0973 | NMY233_0977 | putative lipoprotein | 99.9 | 20 |
| NMY220_0984 | NMY233_0988 | putative lipoprotein | 99.2 | 20 |
| NMY220_0989 | NMY233_0992 | gamma-glutamyltransferase | 99.1 | 20 |
| NMY220_0991 | NMY233_0994 | fructose-1,6-bisphosphatase | 97.7 | 20 |
| NMY220_1050 | NMY233_1054 | putative lipoprotein | 92.4 | 9 |
| NMY220_1067 | NMY233_1070 | putative lipoprotein | 98.9 | 20 |
| NMY220_1069 | NMY233_1072 | CsgG family protein | 98.2 | 20 |
| NMY220_1225 | NMY233_1212 | creA protein | 98.5 | 20 |
| NMY220_1363 | NMY233_1342 | LysM domain/M23 peptidase domain protein | 96.4 | 20 |
| NMY220_1397 | NMY233_1375 | putative lipoprotein | 91.4 | 20 |
| NMY220_1399 | NMY233_1377 | outer membrane autotransporter^3^ | 71.0 | 20 |
| NMY220_1407 | NMY233_1385 | Azurin | 98.9 | 20 |
| NMY220_1447 | NMY233_1426 | integral membrane protein | 94.4 | 20 |
| NMY220_1457 | NMY233_1433 | putative membrane protein | 99.5 | 20 |
| NMY220_1458 | NMY233_1434 | putative periplasmic putrescene-binding ABC transporter, | 99.5 | 20 |
| NMY220_1608 | NMY233_1584 | type IV pilus assembly protein PilP | 97.9 | 20 |
| NMY220_1651 | NMY233_1628 | carbamoyl-phosphate synthase, large subunit | 99.5 | 20 |
| NMY220_1665 | NMY233_1641 | factor H binding protein | 85.4 | 20 |
| NMY220_1674 | NMY233_1650 | putative ABC transporter, periplasmic iron-binding protein | 99.7 | 20 |
| NMY220_1690 | NMY233_1663 | lipoprotein Mlp | 98.7 | 20 |
| NMY220_1732 | NMY233_1707 | putative lipoprotein | 99.4 | 15 |
| NMY220_1738 | NMY233_1713 | transglycosylase SLT domain protein | 97.9 | 19 |
| NMY220_1767 | NMY233_1742 | putative lipoprotein | 97.5 | 20 |
| NMY220_1779 | NMY233_1755 | iron chelate ABC transporter, periplasmic iron chelate-binding protein^3^ | 97.9 | 20 |
| NMY220_1781 | NMY233_1757 | iron chelate ABC transporter, permease protein | 97.7 | 20 |
| NMY220_1876 | NMY233_1855 | phospholipid-binding domain protein | 99.8 | 20 |
| NMY220_1888 | NMY233_1867 | putative lipoprotein, MafA family | 98.5 | 17 |
| NMY220_1914 | NMY233_1891 | phospholipase, patatin family | 99.5 | 20 |

^1^ % amino acid identity across 22 sequenced genomes (includes NM220 and NM233)

^2^ Number of sequenced genomes in addition to NM220 and NM233 containing this locus

^3^ Identical nucleotide sequence, leading gap
