# Supplementary material for: Whole Genome Sequencing to Investigate the Emergence of Clonal Complex 23 Neisseria meningitidis Serogroup Y Disease in the United States
Source: PLoS One. 2012 Apr 27;7(4):e35699. doi: 10.1371/journal.pone.0035699 (PMC3338715; doi:10.1371/journal.pone.0035699)
Supplement: Table S3 — Early clone (NM220) and late clone (NM233) differences in predicted outer membrane proteins, lipoproteins, proteins involved in pilus biogenesis and non-lipoproteins involved in iron acquisition and uptake. (DOCX) [file pone.0035699.s008.docx]

Table S3. Early strain type (NM220) and late strain type (NM233) differences in predicted outer membrane proteins, lipoproteins, proteins involved in pilus biogenesis and non-lipoproteins involved in iron acquisition and uptake.

| Early Clone | Late Clone | Protein | Nucleotide Identity % | Amino Acid Identity % | Amino Acid  Differences^1^ | π_S_^2^ | π_N_ ^3^ |  |
| --- | --- | --- | --- | --- | --- | --- | --- | --- |
| Outer Membrane Proteins | | | | | | | |  |
| NMY220_1312 | NMY233_1295 | PorA | 98.7 | 98.2 | 6c, 3s, 4n, 1 indel | 0.0410 | 0.0210 |  |
| NMY220_1828 | NMY233_1807 | PorB | 83.3 | 79.7 | 43c, 39s, 33n, 32 in 7 indels | 1.2413 | 0.2789 |  |
| NMY220_1777 | NMY233_1754 | FetA | 97.2 | 97.1 | 17c, 12s, 10n, 3 aa in 2 indels | 0.1414 | 0.0321 |  |
| Lipoproteins | | | | | | | |  |
| NMY220_0139 | NMY233_0121 | putative lipoprotein | 99.9 | 99.9 | 1sc | 0.0059 | 0.0015 |  |
| NMY220_0394 | NMY233_0376 | FrpC operon protein | 97 | 96 | 5c, 1s, 4n, 2 indel | 0.0471 | 0.0289 |  |
| NMY220_0407 | NMY233_0389 | putative lipoprotein, MafA family | 99.4 | 99.2 | 1c, 2s, 2n | 0.0410 | 0.0081 |  |
| NMY220_0488 | NMY233_0466 | putative periplasmic putrescene-binding ABC transporter | 99.3 | 100 | - | 0.0577 | 0.0001 |  |
| NMY220_0691 | NMY233_0667 | competence lipoprotein ComL | 98.8 | 100 | - | 0.0924 | 0.0001 |  |
| NMY220_0695 | NMY233_0671 | rare lipoprotein B family | 99.5 | 99.7 | 1 c | 0.0323 | 0.0029 |  |
| NMY220_0734 | NMY233_0710 | putative lipoprotein | 99.9 | 99.7 | 1 n | 0 | 0.0026 |  |
| NMY220_0845 | NMY233_0833 | outer membrane lipoprotein LolB | 99.5 | 99.2 | 1c, 2n | 0.0168 | 0.0078 |  |
| NMY220_0874 | NMY233_0864 | putative lipoprotein | 99.8 | 99.7 | 1c, 1s | 0.0117 | 0.0021 |  |
| NMY220_0884 | NMY233_0874 | putative lipoprotein | 99.7 | 99.5 | 1c, 1s, 1n | 0.0041 | 0.0069 |  |
| NMY220_0959 | NMY233_0945 | sulfate ABC transporter, periplasmic sulfate-binding protein | 99.9 | 99.9 | 1 sc | 0.0056 | 0.0012 |  |
| NMY220_1169 | NMY233_1154 | long-chain-fatty-acid--CoA ligase homolog | 99.7 | 99.7 | 2s | 0.0144 | 0.0027 |  |
| NMY220_1315 | NMY233_1298 | NlpC/P60 family protein | 99.7 | 99.4 | 2c | 0.0098 | 0.0052 |  |
| NMY220_1316 | NMY233_1299 | phospholipase domain protein | 99.6 | 99.2 | 4c, 4n | 0.0173 | 0.0063 |  |
| NMY220_1322 | NMY233_1305 | putative lipoprotein | 97.8 | 97.7 | 7c, 2 s, 2n | 0.1379 | 0.0208 |  |
| NMY220_1351 | NMY233_1331 | putative lipoprotein Ag473^4^ | 94.1 | 93.9 | 1s, 14aa indel | 0.0001 | 0.0059 |  |
| NMY220_1369 | NMY233_1347 | putative lipoprotein | 98.2 | 96.9 | 4c, 3s, 1n | 0.0572 | 0.0314 |  |
| NMY220_1413 | NMY233_1391 | lactoferrin-binding protein | 91.1 | 86.5 | 85c, 40 s, 47 n, 31 aa in 8 indels | 0.2387 | 0.1559 |  |
| NMY220_1427 | NMY233_1405 | peptidyl-prolyl cis-trans isomerase | 98.9 | 98.7 | 2c, 1s, 4aa in 1 indel | 0.0174 | 0.0049 |  |
| NMY220_1438 | NMY233_1416 | antioxidant, AhpC/TSA family | 99.8 | 99.5 | 2 s | 0.0048 | 0.0047 |  |
| NMY220_1472 | NMY233_1449 | periplasmic amino acid-binding ABC transporter | 99.9 | 100 | - | 0.0044 | 0 |  |
| NMY220_1479 | NMY233_1456 | muramoyltetrapeptide carboxypeptidase | 98.8 | 99.1 | 4c, 2s, 1n | 0.0662 | 0.0086 |  |
| NMY220_1482 | NMY233_1459 | copper-containing nitrite reductase, | 99.9 | 99.9 | 1 n | 0.0029 | 0.0013 |  |
| NMY220_1519 | NMY233_1495 | putative lipoprotein | 98.8 | 99.1 | 2c, 1n | 0.0875 | 0.0076 |  |
| NMY220_1521 | NMY233_1497 | GDSL lipase/acylhydrolase domain protein | 99.1 | 99.3 | 2c, 1n | 0.0567 | 0.0064 |  |
| NMY220_1557 | NMY233_1533 | membrane fusion protein MtrC | 99.1 | 99.2 | 2c, 4s | 0.0370 | 0.0101 |  |
| NMY220_1735 | NMY233_1710 | D-methionine ABC transporter, periplasmic D-methionine-binding protein GNA1946 | 99.9 | 99.8 | 1s | 0 | 0.0015 |  |
| NMY220_1759 | NMY233_1734 | autotransporter serine protease | 99.8 | 99.7 | 4c, 1s, 2 aa in 1 indel | 0.0056 | 0.0022 |  |
| NMY220_1908 | NMY233_1885 | Neisserial heparin binding antigen NHBA | 99.9 | 99.8 | 1c, 1s | 0.0023 | 0.0024 |  |
| NMY220_1939 | NMY233_1910 | haemoglobin-haptoglobin-utilization protein A HpuA | 93.0 | 91.0 | 8c, 9s, 11n, 20 aa in 4 indels (17,1,1,1) | 0.1480 | 0.0441 |  |
| Proteins involved in Pilin Structure, Glycosylation, and Assembly | | | | | | | |  |
| NMY220_0259 | NMY233_0241 | pilin glycosyltransferase PglA | 99.9 | 100 | - | 0.0089 | 0 |  |
| NMY220_0858 | NMY233_0847 | Tfp biogenesis protein PilH | 95.3 | 95.1 | 8c, 6s, 7 n | 0.2852 | 0.061 |  |
| NMY220_0859 | NMY233_0848 | Tfp biogenesis protein PilI | 96.3 | 95.6 | 6c, 2s, 2n, 9 aa indel | 0.0643 | 0.0262 |  |
| NMY220_0860 | NMY233_0849 | Tfp biogenesis protein PilJ | 95.2 | 92.5 | 17c, 13s, 11nc, 4 aa indel | 0.0821 | 0.098 |  |
| NMY220_0862 | NMY233_0851 | Minor pilin PilX | 99.9 | 99.7 | 1s | 0 | 0.0026 |  |
| NMY220_1609 | NMY233_1585 | type IV pilus secretin PilQ | 99.9 | 99.9 | 1 c, 1aa indel | 0.0019 | 0.0006 |  |
| NMY220 *pilC1*^4^ | NMY233_1622 | type IV pilus-associated protein PilC1 | 90 | 84 | 69c, 50s, 38n, 13 aa in 9 indels | 0.1067 | 0.0968 |  |
| NMY220  *pilC2*^5^ | absent | type IV pilus-associated protein PilC2 | - | - | - | - | - |  |
| NMY220_0548 | NMY233_0521 | type IV pilus modification protein PilV | 94.4 | 91.5 | 11c, 9s, 2n | 0.326 | 0.0921 |  |
| NMY220_1618 | NMY233_1594 | pilin glycosylation protein PglB | 76 | 72 | - | - | - |  |
| NMY220_1619 | NMY233_1596 | pilin glycosylation protein PglC | 96.7 | 97.4 | 6c, 4s, 1n | 0.1543 | 0.0178 |  |
| NMY220_1620 | NMY233_1597 | pilin glycosylation protein PglD | 99.9 | 99.9 | 1c | 0 | 0.0007 |  |
| NMY220_0624 | NMY233_0600 | pilin glycosylation protein PglE ^6^ | 95.8 | 95.1 | 9c, 4s, 11n, 20aa in 2 indels | 0.0718 | 0.0242 |  |
| NMY220_1617 | NMY233_1593 | pilin glycosylation protein PglF | 99.6 | 99.6 | 1c, 2s | 0.0227 | 0.0034 |  |
| NMY220_1616 | NMY233_1592 | pilin glycosylation protein  PglG | 99.8 | 99.9 | 1 n | 0.0208 | 0.0012 |  |
| NMY220_1615 | NMY233_1591 | pilin glycosylation protein PglH | 98.9 | 99.2 | 3c, 3s, 2n | 0.0522 | 0.0081 |  |
| NMY220_1635 | NMY233_1612 | pilin glycosylation protein PglI | 97.7 | 97.7 | 15c, 3s, 8n, 3 aa indel | 0.0767 | 0.0203 |  |
| NMY220_0598 | NMY233_0572 | pilin glycosylation protein PglL | 99.0 | 98.8 | 11c, 4sc | 0.0539 | 0.011 |  |
| Non-lipoprotein Iron Acquisition and Uptake Proteins | | | | | | | | |
| NMY220_0487 | NMY233_0465 | Transferrin binding protein A (*tbpA*) | 98.7 | 98.7 | 10c, 5s, 5n, 3aa indel | 0.0544 | 0.0133 |  |
| NMY220_0486 | NMY233_0464 | Transferrin binding protein B (*tbpB*) | 97.7 | 96.8 | 15c, 15s, 10n, 5 aa in 3 indel | 0.0750 | 0.0320 |  |
| NMY220_1412 | NMY233_1390 | Lactoferrin binding protein A (*lbpA*) | 98.3 | 98.2 | 15c, 4s, 14n, 1 aa indel | 0.0783 | 0.0222 |  |

^1^c, conservative amino acid substitution; s, semi-conservative amino acid substitution; n, non-conservative amino acid substitution; aa, amino acid

^2^ π_S_, number of synonymous changes per synonymous site

^3^ π_N_, number of non-synonymous changes per non-synonymous site

^4^GenBank Accession number JN681261

^5^GenBank Accession number JN681262

^6^Tandem repeat difference
