# Supplementary material for: Whole Genome Sequencing to Investigate the Emergence of Clonal Complex 23 Neisseria meningitidis Serogroup Y Disease in the United States
Source: PLoS One. 2012 Apr 27;7(4):e35699. doi: 10.1371/journal.pone.0035699 (PMC3338715; doi:10.1371/journal.pone.0035699)
Supplement: Table S4 — Proteins encoded by genes found in only one of the clones. (DOCX) [file pone.0035699.s009.docx]

Table S4. Proteins encoded by genes found in only one of the strain types.

| NMY220 | | | NMY233 | | |
| --- | --- | --- | --- | --- | --- |
| Gene | **Protein** | **Length^1^** | **Gene** | **Protein** | **Length^1^** |
| NMY220_0312 | conserved hypothetical protein | 137 | NMY233_0596 | ZitB | 322 |
| NMY220_0399 | hypothetical protein | 98 | NMY233_0815 | hypothetical protein | 59 |
| NMY220_0517 | hypothetical protein | 206 | NMY233_0816 | hypothetical protein | 65 |
| NMY220_0518 | hypothetical protein | 312 | NMY233_0818 | conserved hypothetical protein | 122 |
| NMY220_0834 | hypothetical protein | 96 | NMY233_0819 | conserved hypothetical protein | 182 |
| NMY220_0835 | conserved hypothetical protein | 63 | NMY233_0823 | conserved hypothetical protein | 130 |
| NMY220_0836 | putative integral membrane protein | 135 | NMY233_0824 | conserved domain protein | 148 |
| NMY220_0919 | conserved hypothetical protein | 106 | NMY233_0921 | rubredoxin | 57 |
| NMY220_0952 | membrane protein | 161 | NMY233_1280 | FrpC operon protein | 230 |
| NMY220_1368 | conserved hypothetical protein | 110 | NMY233_1732 | conserved hypothetical protein | 110 |
| NMY220_1648 | conserved hypothetical protein | 114 | NMY233_1744 | putative membrane protein | 339 |
| NMY220_1901 | conserved hypothetical protein | 118 | NMY233_0822 | putative membrane protein | 97 |
| NMY220_1292 | hypothetical protein | 145 | NMY233_0820 | putative lipoprotein | 95 |
| NMY220_1804 | hypothetical protein | 66 | NMY233_0821 | hypothetical protein | 57 |
| NMY220_1946 | putative membrane protein | 203 | NMY233_1921 | putative transmembrane transport protein | 265 |
| NMY220_1932 | hypothetical protein | 82 | NMY233_1911 | hypothetical protein | 62 |
| NMY220_1933 | hypothetical protein | 38 |  |  |  |
| NMY220_1941 | hypothetical protein | 96 |  |  |  |
| NMY220_1947 | putative membrane protein | 112 |  |  |  |

^1^In amino acids
